# Influence of HeartMate 3™ on Bispectral Index™ monitor: a retrospective observational study

**DOI:** 10.1007/s10877-025-01272-4

**Published:** 2025-03-06

**Authors:** Seiichi Azuma, Masaaki Asamoto, Shinichi Akabane, Mariko Ezaka, Mikiya Otsuji, Kanji Uchida

**Affiliations:** 1 Department of Anesthesiology and Pain Relief Center, The University of Tokyo Hospital, Hongo 7-3-1, Bunkyo-ku, Tokyo, 113-8655 Japan; 2 Graduate School of Medicine, The University of Tokyo, Tokyo, Japan; 3 Department of Anesthesiology, Tokyo Teishin Hospital, Tokyo, Japan

**Keywords:** Artifacts, Bispectral index, Electroencephalogram, Electromyographic index, HeartMate 3, Left ventricular assist device

## Abstract

Electroencephalogram-derived monitors are affected by various artifacts. HeartMate 3™ operates at frequency bands that overlap with those used for calculating the electromyographic index (EMG) and bispectral index (BIS) on the Bispectral Index™ monitor. This study investigated whether HeartMate 3 elevates these values, a change not predicted with HeartMate II™. This retrospective observational study included data from patients who underwent HeartMate 3 or II implantation between April 2008 and December 2023 as extracted from our institutional database. Patient-wise median EMG and BIS were compared between the pre-LVAD period (from the start of surgery to initiation of cardiopulmonary bypass) and the post-LVAD period (from the end of cardiopulmonary bypass to end of surgery). Data were obtained from 33 and 43 patients who underwent HeartMate 3 and HeartMate II implantation, respectively. Patients with HeartMate 3 implantation showed significant elevation in the EMG (pre-LVAD, mean ± standard deviation, 26.1 ± 1.0 dB; post-LVAD, 39.5 ± 2.8 dB; *P* < 0.001) without a significant change in the BIS (pre-LVAD, 44.5 ± 8.1; post-LVAD, 45.5 ± 7.1; *P* = 0.35). In contrast, patients with HeartMate II implantation did not show significant changes in either the EMG (pre-LVAD, 26.1 ± 1.2 dB; post-LVAD, 27.1 ± 4.1 dB; *P* = 0.16) or BIS (pre-LVAD, 45.1 ± 9.2; post-LVAD, 43.0 ± 8.1; *P* = 0.071). HeartMate 3 significantly elevates EMG. Anesthesiologists should be aware of this to appropriately interpret EMG elevation in patients with HeartMate 3.

*Trial registration*: Japan Registry for Clinical Trials identifier: jRCT1030230549 (date of registration: January 10, 2024.

## Introduction

Anesthesiologists frequently use electroencephalogram (EEG)-derived monitors to assess the depth of anesthesia and provide a quantitative measure of the effects of anesthetic agents [1]. Considering their critical roles in preventing excessive anesthesia and intraoperative awareness, it is essential to understand the influence of various artifacts on these monitors. Previous studies have reported that left ventricular assist devices (LVADs) induce specific artifacts on electrocardiogram (ECG) [2, 3], thereby raising concerns about their contamination of EEG signals and their influence on EEG-derived monitors.

**Fig. 1:**
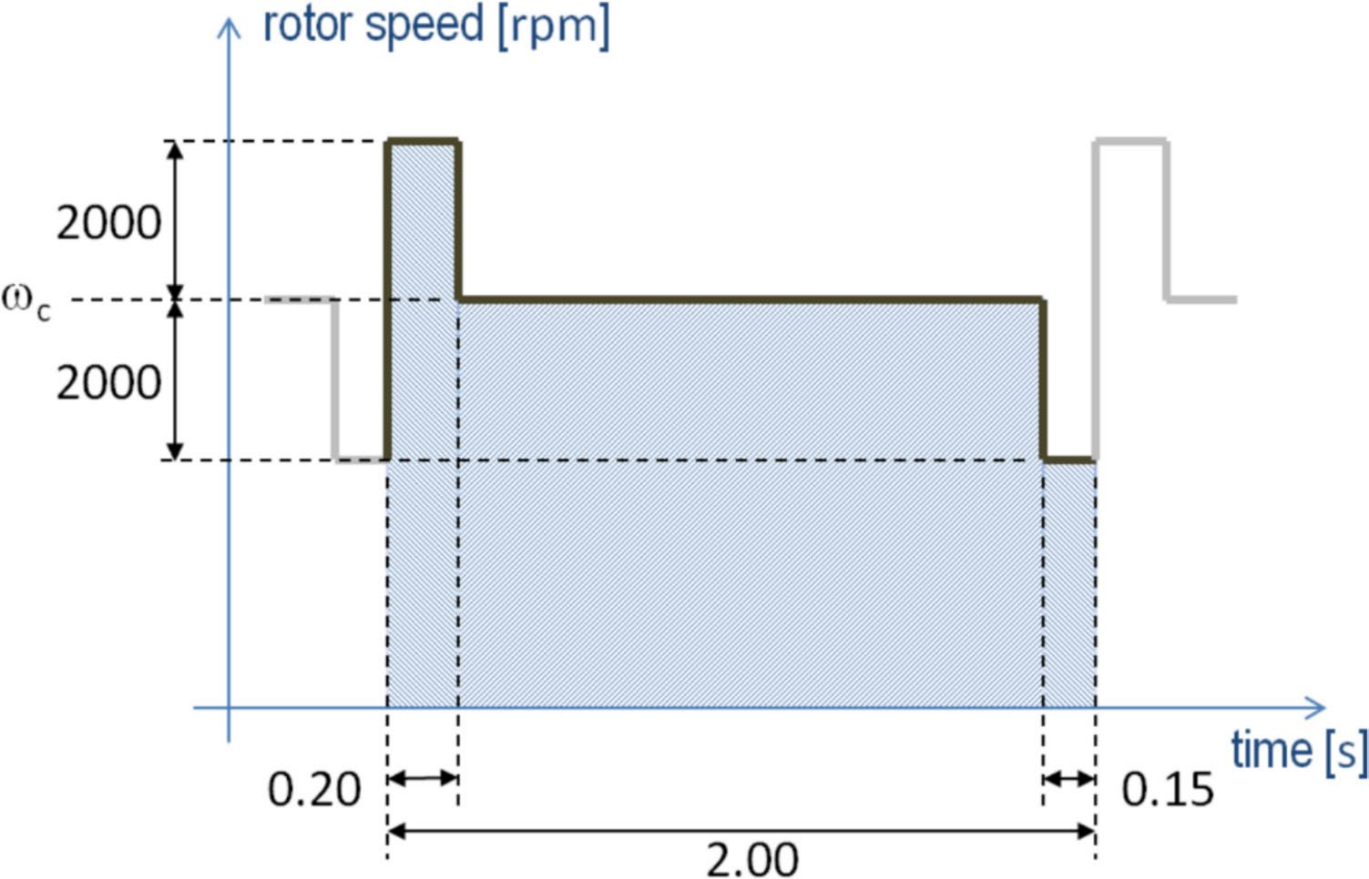
HeartMate 3™ left ventricular assist system rotor speed chart, showing the repeating 2-s cycle. The cycle begins with the rotor speed operating at 2000 rpm above the set rotor speed (${\omega }_{c}$) for 0.20 s, followed by operation at the set speed, and ends with 0.15 s at 2000 rpm below the set value. HeartMate 3 is a trademark of Abbott or its related companies. Reproduced with permission of Abbott, © 2024. All rights reserved. Abbreviation: *rpm* revolutions per minute

Currently, HeartMate 3™ (Abbott, Abbott Park, Illinois, USA) is one of the most commonly used LVADs, operating at approximately 5000 revolutions per minute (rpm) [4]. At ≥ 4000 rpm, it generates artificial pulses by repeating a 2-s cycle, operating at the set rotational speed for most of the cycle, at 2000 rpm below the set value for 0.15 s, and at 2000 rpm above the set value for 0.2 s (Fig. 1). Such rotations induce specific electromagnetic artifacts at corresponding frequencies, which are observed as one principal peak and two minor peaks in the ECG spectrum [3]. Importantly, these frequencies overlap with the frequency band used to calculate the electromyographic index (EMG) and bispectral index (BIS) on the Bispectral Index™ (BIS™) monitor (Medtronic, Dublin, Ireland). The principal peak of HeartMate 3 operating at 4200–6600 rpm lies within the 70–110-Hz frequency band, which is used to calculate the EMG, as outlined in the instructions for use of the BIS monitor (Pharmaceuticals and Medical Devices Agency, approval number 22400BZX00507000). The lower minor peak of HeartMate 3 operating at ≤ 4800 rpm lies below 47 Hz, a frequency range crucial for calculating the BIS [5]. In contrast, the older HeartMate II™ model (Abbott, Abbott Park, Illinois, USA) operates at approximately 9000 rpm [4], corresponding to a much higher frequency than that used by the BIS monitor.

Therefore, HeartMate 3 may influence both the EMG and BIS through a mechanism that does not apply to HeartMate II. In this study, we aimed to clarify the impact of HeartMate 3 on the EMG and BIS to enhance the quality of anesthetic management through appropriate interpretation of these measurements in patients with HeartMate 3 implantation.

## Methods

### Data acquisition

This retrospective observational study was conducted as part of the comprehensive research project “Examining Optimal Physiological Parameters for Ideal Perioperative Management Utilizing Medical Records in the Department of Anesthesiology and Pain Relief Center,” approved by the Research Ethical Committee of the Faculty of Medicine at the University of Tokyo, Tokyo, Japan (approval number: 2023166NI, date of approval: October 2, 2021) and registered in the Japan Registry for Clinical Trials (registration number: jRCT1030230549, date of registration: January 10, 2024). No sample size calculation was performed, and we used as much data as available. The requirement for obtaining written informed consent was waived by the committee. This manuscript adheres to the STROBE guidelines.

This study included data from patients who underwent HeartMate 3 or HeartMate II implantation as their first LVAD at the University of Tokyo Hospital between April 2008 and December 2023. Data were extracted by searching the anesthetic record database and excluding the LVAD conversion cases. A BIS monitor was routinely used during these procedures. Instantaneous EMG and BIS values, calculated by proprietary algorithms of the BIS monitor, were retrospectively collected at 1-min intervals from the anesthetic record database for patients who underwent surgery before March 2021 and at 3-s intervals from the exported records of the bedside monitor for patients who underwent surgery after April 2021. Demographic data, including the final rotational speeds of the LVADs, were obtained from medical records.

The ECG and EEG waveform data were acquired from the exported records of the bedside monitor with a resolution of 250 Hz for patients undergoing implantation after April 2021. Several technical problems should be noted for interpreting these data. First, the waveform data were filtered according to the display settings selected by the attending anesthesiologists before export. The filters included an optional hum filter and one of the bandpass filters (1–18, 0.3–40, 0.05–18, or 0.05–150 Hz) for ECG, as well as an optional unspecified filter for EEG. Second, the values of the EEG waveform at every other point systematically matched their neighboring points, a phenomenon not seen in ECG data, suggesting that it had been upsampled to 250 Hz from an effective resolution of 125 Hz. Considering that the 70–110 Hz band was of interest, we retained the exported 250 Hz sampling rate for frequency analysis despite recognizing aliasing at 62.5 Hz. Consequently, power at $f$ Hz appeared at both $f$ Hz and $125-f$ Hz in the EEG frequency analysis owing to aliasing effects.

### Analysis

The pre-LVAD period was defined as the interval from the start of surgery to initiation of cardiopulmonary bypass, whereas the post-LVAD period was defined as the interval from the end of cardiopulmonary bypass to end of surgery. The median EMG and BIS were calculated for the pre- and post-LVAD periods for each patient. Subsequently, these median values were compared between the pre- and post-LVAD periods for HeartMate 3 and II to investigate their influence on the EMG and BIS. Inasmuch as the influence on the BIS was specifically anticipated when the rotational speed was ≤ 4800 rpm, a comparison restricted to patients with a final rotational speed of ≤ 4800 rpm was performed.

ECG and EEG power spectral densities were calculated to explore the artifacts in detail using the multitaper method [6, 7] with the following parameters: window length of 2048 (corresponding to 8.192 s), offset of 4.096 s, time half-bandwidth NW of 3, and number of tapers k of 6, providing a spectral resolution of 0.73 Hz. These were displayed as spectrograms on the dB scale (i.e., 10 times the base-10 logarithm of the power spectral density on the μV^2^/Hz scale) together with trends in the EMG and BIS to visualize their relationships. The EEG spectrogram was annotated at times and frequencies where peaks were observed on ECG to evaluate the correspondence of peaks between ECG and EEG.

### Statistical analysis

Continuous data are expressed as mean ± standard deviation. Paired *t*-tests were performed to compare the EMG and BIS between the pre- and post-LVAD periods within each model (HeartMate 3 or HeartMate II). Statistical significance was set at *P* < 0.05 (two-sided). Python (version 3.11.6, Python Software Foundation, https://www.python.org/) was used for all analyses.

## Results

Data from 33 and 43 patients who underwent HeartMate 3 and HeartMate II implantation between 2019 and 2023 and between 2010 and 2019, respectively, were obtained. The final rotational speeds of HeartMate 3 and HeartMate II were 4964 ± 309 and 8391 ± 265 rpm, respectively (*P* < 0.001). There were no significant differences in age, sex, and pathology between the groups (Table 1). Details of the anesthetic management for these procedures are provided in Appendix 1.

**Table 1:** Demographic data of the patients

	HeartMate 3™	HeartMate II™	*P* value
Age (years)	44.8 ± 15.3	44.1 ± 12.6	0.85
Sex			0.079
Female	9 (27.3%)	4 (9.3%)	
Male	24 (72.7%)	39 (90.7%)	
Height (cm)	168.1 ± 9.5	169.7 ± 5.5	0.39
Weight (kg)	63.5 ± 15.8	60.4 ± 7.7	0.30
BMI (kg/m^2^)	22.3 ± 3.8	21.0 ± 2.7	0.11
Pathology			0.49
DCM	24 (72.7%)	37 (86.1%)	
ICM	4 (12.1%)	2 (4.7%)	
HCM	2 (6.1%)	3 (7.0%)	
Sarcoidosis	1 (3.0%)	1 (2.3%)	
GCM	1 (3.0%)	0 (0.0%)	
ARVC	1 (3.0%)	0 (0.0%)	
Use of ICD	2 (6.1%)	4 (9.3%)	0.93
Use of CRT-P	0 (0.0%)	1 (2.3%)	1.0
Use of CRT-D	14 (42.4%)	22 (51.2%)	0.60
Use of IABP	9 (27.3%)	17 (39.5%)	0.38

Data are presented as mean ± standard deviation for continuous variables or as numbers (percentages) for discrete variables. *P* values were calculated using Welch’s *t-*test for continuous variables and the chi-square test for discrete variables

*BMI* body mass index, *DCM* dilated cardiomyopathy, *ICM* ischemic cardiomyopathy, *HCM* hypertrophic cardiomyopathy, *GCM* giant cell myocarditis, *ARVC* arrhythmogenic right ventricular cardiomyopathy, *ICD* implantable cardioverter defibrillator, *CRT-P* cardiac resynchronization therapy pacemaker, *CRT-D* cardiac resynchronization therapy defibrillator, *IABP* intra-aortic balloon pump

**Fig. 2:**
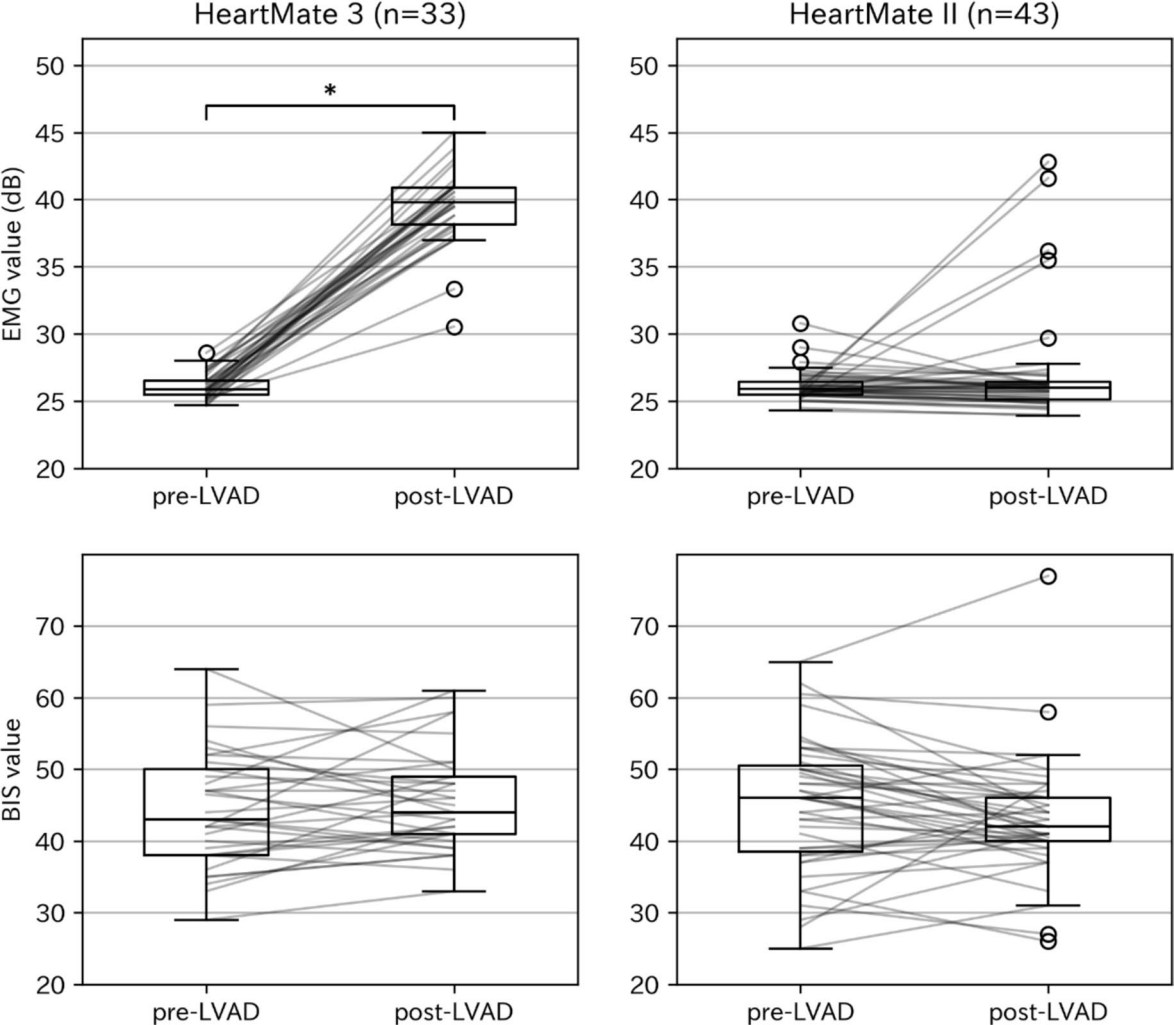
Change in the EMG (top) and BIS (bottom) between the pre- and post-LVAD periods for patients undergoing HeartMate 3™ (left) or HeartMate II™ (right) implantation. Boxes with horizontal lines represent the interquartile ranges with median values. Whiskers extend to the smallest and largest values within 1.5 times the interquartile range, and circles indicate outliers beyond this range. Thin connecting lines link individual pre- and post-LVAD data, illustrating the changes for each patient. The asterisk (*) indicates statistical significance, observed only for the EMG in the HeartMate 3 group (*P* < 0.001, top left). Abbreviations: *BIS* bispectral index, *EMG* electromyographic index, *LVAD* left ventricular assist device

The changes in the EMG and BIS are shown in Fig. 2. Patients with HeartMate 3 implantation had a significantly higher EMG in the post-LVAD period (39.5 ± 2.8 dB) than in the pre-LVAD period (26.1 ± 1.0 dB) (*P* < 0.001), without significant changes in the BIS (pre-LVAD, 44.5 ± 8.1; post-LVAD, 45.5 ± 7.1; *P* = 0.35). The change in the BIS remained non-significant after restricting our comparison to the 11 patients with a final rotational speed of ≤ 4800 rpm (pre-LVAD, 47.3 ± 4.3; post-LVAD, 47.1 ± 7.3; *P* = 0.92). Patients with HeartMate II implantation did not show significant changes in either the EMG (pre-LVAD, 26.1 ± 1.2 dB; post-LVAD, 27.1 ± 4.1 dB; *P* = 0.16) or BIS (pre-LVAD, 45.1 ± 9.2; post-LVAD, 43.0 ± 8.1; *P* = 0.071).

**Fig. 3:**
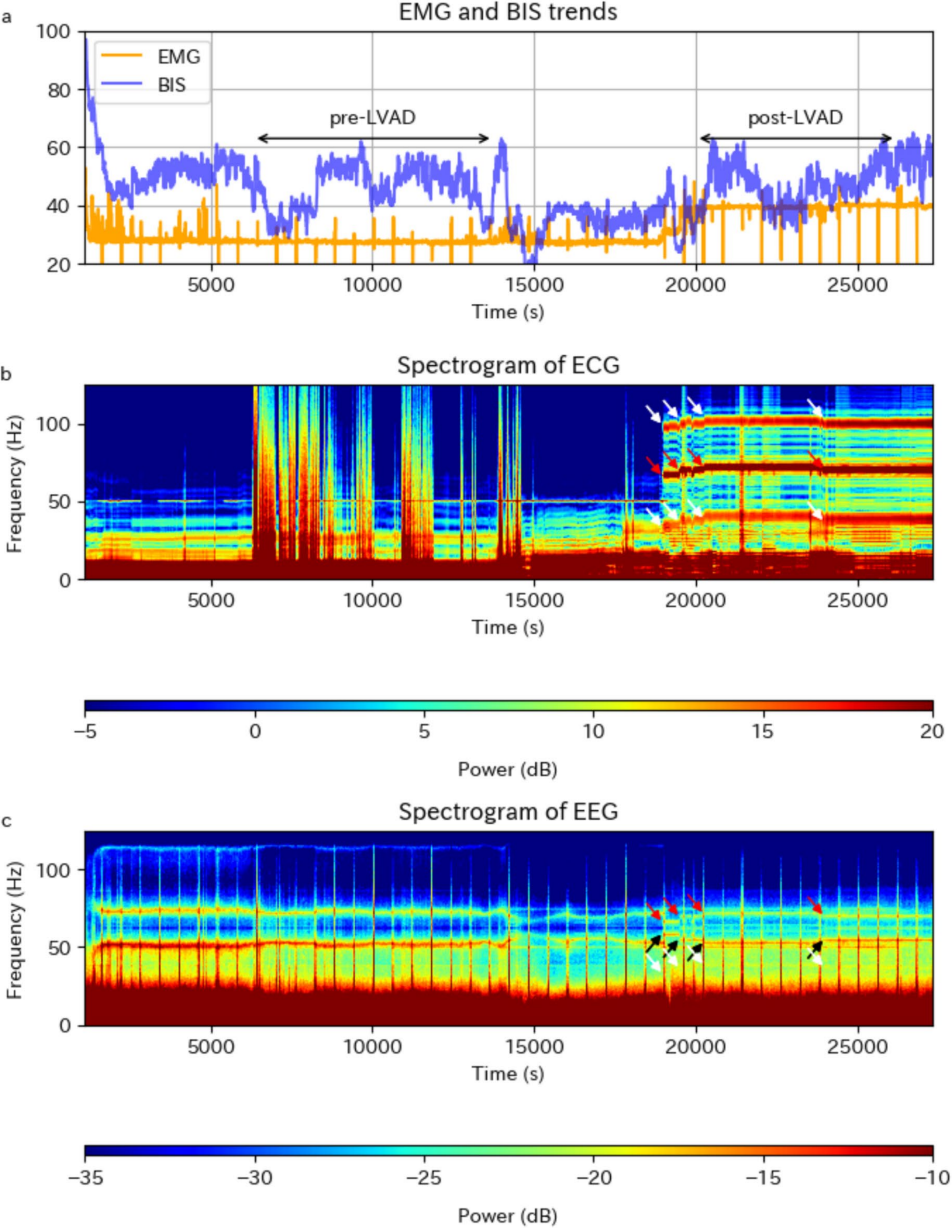
Trends in the EMG and BIS and ECG and EEG spectrograms of a patient undergoing HeartMate 3™ implantation. **a** Trends in the EMG (orange) and BIS (blue), showing elevated EMG in the post-LVAD period. Periodic sharp changes in the EMG were due to automatic impedance checks performed at 10-min intervals. **b** The ECG spectrogram of the same patient, showing a principal peak around 70 Hz (red arrows) and two minor peaks (white arrows, at ± 33 Hz from red arrows) appearing in the post-LVAD period. **c** The EEG spectrogram of the same patient. Peaks were observed at the same time and frequency as the principal peak on ECG (red arrows) and its aliasing frequency (black arrows, at 125 Hz minus frequencies at red arrows), which can be distinguished from the hum noise at 50 Hz and its aliasing artifacts at 75 Hz. A peak corresponding to the lower minor peak on ECG (white arrows, at 33 Hz below red arrows) was also observed. Abbreviations: *BIS* bispectral index, *EMG* electromyographic index; *ECG* electrocardiogram, *EEG* electroencephalogram, *LVAD* left ventricular assist device

**Fig. 4:**
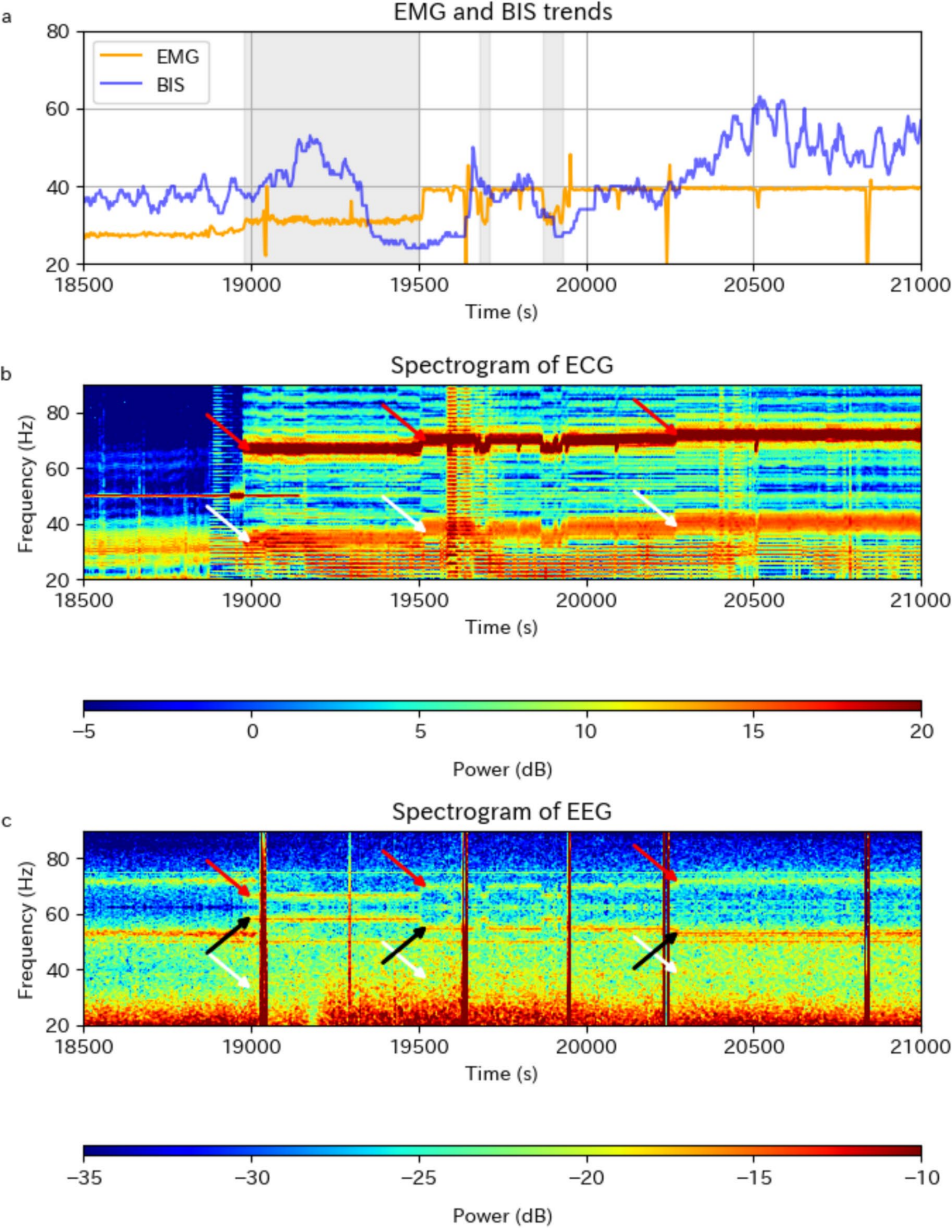
Detailed trends in the EMG and BIS with ECG and EEG spectrograms around the onset of EMG elevation in the same patient as shown in Fig. 3. **a** Trends in the EMG (orange) and BIS (blue), focusing on the onset of EMG elevation. Gray shaded areas indicate periods where EMG elevation was mild. Periodic sharp changes in EMG were due to automatic impedance checks performed at 10-min intervals. **b** The ECG spectrogram of the same patient, focusing on the beginning of the principal peak (red arrows) and the lower minor peak (white arrows, at 33 Hz below the red arrows). The principal peak shows lower frequencies during periods when the EMG elevation was mild. **c** The EEG spectrogram of the same patient with the same frequency band as that of ECG. Peaks corresponding to the principal peak on ECG (red arrows) and its aliasing artifacts (black arrows, at 125 Hz minus frequencies at red arrows) show an identical trend in their frequencies as seen on ECG, whereas a similar correspondence for the lower minor peak (white arrows, at 33 Hz below the red arrows) is unclear. The artifacts at 50 and 75 Hz are likely the hum noise and its aliasing, respectively, whereas the source of the artifacts at 53 and 72 Hz before EMG elevation was unspecified. Abbreviations: *BIS* bispectral index, *EMG* electromyographic index, *ECG* electrocardiogram, *EEG* electroencephalogram, *LVAD* left ventricular assist device

ECG and EEG waveform data were obtained from 16 patients who underwent HeartMate 3 implantation after April 2021. Eight patients showed one principal peak and two minor peaks at ± 33 Hz from the principal peak on the ECG throughout the post-LVAD period (Fig. 3b). The remaining eight patients only showed the principal peak and lower minor peak, likely due to the application of a narrower bandpass filter for the ECG. The principal peak appeared predominantly at 70–90 Hz, concurrent with elevated EMG. Furthermore, EMG elevation was mild during periods when the principal peak was below 70 Hz (Fig. 4a, b). No association was observed between the change in the frequency of the principal peak and the BIS.

In nine of the 16 patients mentioned above, the EEG spectrogram showed a peak at the same time and frequency as the principal peak on the ECG (Fig. 3c). A clear match in the frequency trends between EEG and ECG was observed (Fig. 4b, c). Two additional patients, with a relatively mild EMG elevation, shown as outliers in Fig. 2, did not have a visible EEG peak corresponding to the principal peak on the ECG. The presence of an EEG peak corresponding to the principal peak on ECG was difficult to evaluate owing to the aliasing artifacts of the hum noise or other unspecified artifacts at similar frequencies for the remaining five patients. While some patients exhibited a peak on EEG at the same time and frequency as the lower minor peak on ECG, their correspondence was not definitive because of the relatively weak signal and lack of clear match in the frequency trend between EEG and ECG (Fig. 4b, c). In addition to these peaks in the post-LVAD period, various other artifacts were observed on EEG (Fig. 3c).

## Discussion

We demonstrated significant EMG elevation after HeartMate 3 implantation, but not after HeartMate II implantation. This suggests that EMG elevation is caused specifically by HeartMate 3. Furthermore, we identified characteristic artifacts on the EEG after HeartMate 3 implantation, directly indicating the source of EMG elevation and highlighting the substantial interference of HeartMate 3 with EEG-derived monitors.

The characteristic artifacts on the EEG matched the frequency trend of the principal peak on the ECG, indicating a common source of these artifacts. The principal peak observed on ECG, which was accompanied by two minor peaks flanking it at ± 33 Hz, is most likely attributable to HeartMate 3 because the pattern was similar to that previously reported [3]. In addition, it is consistent with the device’s operation with pulsatile ± 2000 rpm acceleration and deceleration. Overall, the characteristic EEG artifacts are presumably caused by HeartMate 3.

Moreover, these characteristic artifacts occurred concurrently with EMG elevation. Several observations support a direct relationship between the principal peak and EMG elevation. First, the frequency of the principal peak, which occurred predominantly at 70–90 Hz, was consistent with the 70–110-Hz frequency band used to calculate the EMG. Second, two patients with mild EMG elevation did not show a clear principal peak on the EEG. Finally, periods with the principal peak below 70 Hz corresponded to mild EMG elevation.

In contrast to the relationship between the principal peak and EMG elevation, we could not clarify the effect of the lower minor peak on the BIS, as predicted by the suggested algorithm [5]. We propose three possible interpretations. First, because the lower minor peak is generated for only 0.15 s every 2 s (Fig. 1), the contribution to the spectrum is expected to drop to 7.5% of its original power, corresponding to a decrease of −11 dB. While this drop alone did not prevent its detection on ECG, additional attenuation during transmission to the scalp likely resulted in its uncertain detection on the EEG in this study. Second, even if sufficient power reached the EEG, several concerns regarding its impact on the BIS remain. A suppression ratio of > 10% would reduce the lower minor peak’s contribution to BIS calculation [5]. However, we did not find a sustained suppression ratio of ≥ 10% during the pre- and post-LVAD periods in the 11 patients with a final rotational speed of ≤ 4800 rpm, suggesting that this is unlikely to be a dominant factor. Another consideration is the preprocessing stage. Specifically, epochs with marked changes in voltage variance from the recent average are excluded from further processing, enabling BIS to adapt gradually by discarding sudden fluctuations [8]. However, as described in [5, 8], each epoch lasts 2 s, meaning that it always includes exactly one full cycle of the HeartMate 3 rotational speed change (Fig. 1). Therefore, we speculate that the changes in the rotational speed of HeartMate 3 are not accompanied by inter-epoch variations and are not discarded by the preprocessing as epochs with marked changes, although the actual algorithm remains proprietary. Third, even if the lower minor peak had enough power and it elevated BIS, this change could have been masked by other factors. Unlike the EMG, the BIS was directly affected by anesthetic management and varied considerably within each case, as shown in Fig. 3 and discussed in Appendix 2. The anesthetics typically switched before and after cardiopulmonary bypass (Appendix 1), accompanied by substantial changes in pharmacokinetics and pharmacodynamics [9]. Incorporating these effects into the analysis was extremely challenging.

The importance of observing waveforms or spectrograms, rather than numerical indices such as the BIS, has been emphasized [10–12]. Our study supports this concept in two aspects. First, observing the spectrogram may distinguish EMG elevation caused by muscle activity from that caused by artifacts from HeartMate 3. While the term “EMG” implies the quantification of muscle activity, its evident influence on EEG is observed over a broad frequency range above 20 Hz, not just limited to the 70–110 Hz range [13]. Therefore, muscle activity elevates both the EMG and BIS [14], causing the reported correlations between them [15, 16]. Conversely, artifacts limited to the 70–110 Hz range will elevate the EMG but not the BIS, as observed in this study and a previous one involving the artifacts by electrolytic detachment of endovascular coils [17]. If the EMG is elevated without changes in the broad spectrum above 20 Hz, anesthesiologists may reasonably conclude that it is unlikely attributable to muscle activity. Second, observing the spectrogram may identify the lower minor peak on EEG, which may elevate the BIS. While this effect was not clarified in this study, its occurrence cannot be ruled out, given the variability in the extent of artifact transmission from HeartMate 3 to EEG. Such variability is indicated by the two patients in this study who showed mild EMG elevation and unclear artifacts on EEG, as well as by a previous study reporting variability in the transmission of cardiac activity to the scalp [18].

There were challenges in interpreting the EEG spectrogram in this study. As noted in the Methods section, power at $f$ Hz appeared at both $f$ Hz and $125-f$ Hz in the spectrogram because we used 250 Hz data, which had been upsampled from 125 Hz. Regarding the principal peak, we believe that the peak at a higher frequency represented the real source owing to its clear match with the ECG and concurrent EMG elevation. Notably, there were artifacts on EEG at similar frequencies before EMG elevation (Figs. 3 and 4). We speculate that the artifacts at 70–75 Hz observed before EMG elevation were aliasing from a source at 50–55 Hz due to the lack of EMG elevation, although we could not identify their precise source. Additional artifacts may have been caused by epicardial pacing after cardiopulmonary bypass [19], along with implanted devices and intra-aortic balloon pumping [20] used in some patients (Table 1), although they could not be specifically identified in our dataset. Furthermore, we could not identify the source of the artifacts at approximately 40 Hz during both the pre- and post-LVAD periods (Fig. 3c), making it difficult to determine whether the lower minor peak reached the EEG.

In addition to the interpretation challenges mentioned above, this study has further limitations. First, the comparison between the pre- and post-LVAD periods involved changes in factors other than LVAD, including anesthetic management. Moreover, there were differences in the frequency of ketamine and remimazolam use between the HeartMate 3 and HeartMate II groups (Appendix 1), further complicating the BIS interpretation. Additionally, ketamine has been reported to increase activity up to the 51–99 Hz range [21], which might have influenced EMG calculation although it was only used adjunctively during induction, more than 1 h before the analyzed periods. Second, data quality differed between the groups. While 3-s interval trend and waveform data were available for 16 patients in the HeartMate 3 group, only 1-min interval trend data were available for all patients in the HeartMate 2 group. The use of 1-min interval data may have increased the variability in the analysis. Additionally, the lack of waveform data for the HeartMate II group prevented us from demonstrating the absence of the characteristic artifacts observed with HeartMate 3 in patients with HeartMate II. As a result, the discussion of the underlying mechanism was based on descriptive observations rather than statistical evidence. Third, the proportion of female participants differed between the two groups (9.3% for HeartMate II and 27.3% for HeartMate 3, *P* = 0.079; Table 1). This difference, as well as the differences in the frequency of ketamine and remimazolam mentioned prior, is unlikely to significantly affect our main results but is acknowledged as part of the study’s demographic characteristics. Fourth, our discussion is based on existing descriptions of the BIS [5, 8], while the real algorithm remains proprietary. Finally, the influence on other EEG-derived monitors remains to be studied.

In conclusion, we revealed that HeartMate 3 causes significant EMG elevation, likely through artifacts at its rotational speed. Anesthesiologists should be aware of this to appropriately interpret EMG elevation in patients with HeartMate 3.

## Figures and Tables

**Fig. 5:**
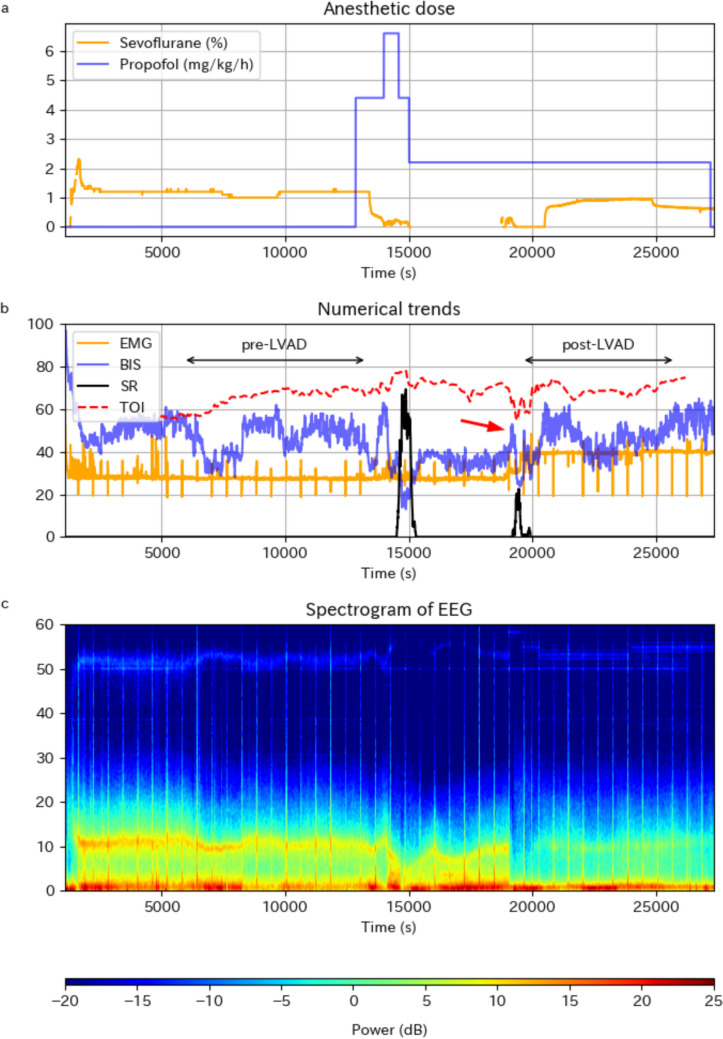
Information related to interpreting the BIS in the same patient as shown in Fig. 3. **a** Trends in the concentration of end-tidal sevoflurane (orange) and infusion rate of propofol (blue). **b** Trends in the EMG (orange), BIS (blue), SR (black), and TOI (red, dashed). The red arrow indicates an increase in BIS around 19,000 s, followed by a decrease, as discussed in the text. **c** The EEG spectrogram, focusing on the frequency band related to anesthetic agents. Abbreviations: *BIS* bispectral index; EMG, electromyographic index, *ECG* electrocardiogram, *EEG* electroencephalogram, *LVAD* left ventricular assist device, *SR* suppression ratio, *TOI* tissue oxygenation index

## Data Availability

The data are not publicly available due to the restricted use of clinical data.

## Appendix 1: Anesthetic management for the procedures

Anesthetic agents used for each period are described below. Given the retrospective nature of this study, the specific reasons for choosing anesthetic agents for each patient could not be determined.

### Induction

Anesthesia was typically induced using midazolam, fentanyl, and either vecuronium or rocuronium. Ketamine was co-administered during induction for four patients undergoing HeartMate 3 implantation and 27 patients undergoing HeartMate II implantation. Six patients undergoing HeartMate 3 implantation received remimazolam instead of midazolam.

### Pre-LVAD period

The pre-LVAD period typically began more than 1 h after induction. During this period, sevoflurane was the most commonly used anesthetic for maintenance, administered to 27 patients undergoing HeartMate 3 implantation and 36 patients undergoing HeartMate II implantation. Six patients undergoing HeartMate 3 implantation continued on remimazolam after induction. Among the remaining patients undergoing HeartMate II implantation, five received propofol, one received desflurane, and one received midazolam.

### Post-LVAD period

For patients initially maintained on sevoflurane or desflurane during the pre-LVAD period, the anesthetic was switched to propofol or midazolam during cardiopulmonary bypass. In the post-LVAD period, sevoflurane was resumed for maintenance in some patients (13 undergoing HeartMate 3 implantation and 21 undergoing HeartMate II implantation), while others were maintained on propofol or a combination of sevoflurane and propofol. Patients maintained on propofol or remimazolam during the pre-LVAD period typically remained on the same anesthetic in the post-LVAD period, except for one case where remimazolam was switched to propofol.

Adequate doses of vecuronium or rocuronium were administered to ensure sufficient muscle relaxation throughout both the pre- and post-LVAD periods.

## Appendix 2: Interpretations of changes in the BIS observed in Fig. 3

Here, we discuss the changes in the BIS as shown in Fig. 3, along with relevant data. This highlights the importance of observing the spectrogram, as discussed in the main text, although it is not the primary focus of this study. Figure 5 presents the anesthetic dose, numerical trends, and EEG spectrogram including delta and alpha frequencies. The numerical trends include the EMG, BIS, and suppression ratio (SR), calculated by the BIS monitor, as well as the tissue oxygenation index (TOI) calculated by the NIRO™ near-infrared spectrometer (Hamamatsu Photonics, Hamamatsu, Japan), which monitors cerebral blood oxygenation.

Decreases in the BIS were correlated with decreases in alpha peak frequency and an increase in delta power on the spectrogram (Fig. 5b, c), as described in the literature [22]. These changes were partly associated with the doses of sevoflurane and propofol doses (Fig. 5a).

An increase in the BIS was observed before the post-LVAD period (Fig. 5b, red arrow). This occurred shortly after the onset of artifacts from HeartMate 3, as indicated by the mild EMG elevation. While this increase in the BIS may have been caused by HeartMate 3, other plausible explanations include increased body temperature or increased hepatic blood flow related to paced or restored cardiac activity with catecholamine after cardiopulmonary bypass, both of which could reduce propofol concentration. Subsequently, the BIS decreased rapidly with a concurrent increase in SR, and the spectrogram showed a rapid decrease in alpha activity. This may be associated with a decrease in cerebral blood flow, as indicated by the decrease in TOI. Further discussion on these changes is beyond the scope of this study.
